# Frailty Trajectories and Their Predictors in Chinese Empty-Nest Older Adults: An 8-Year Longitudinal Study

**DOI:** 10.3390/healthcare14040537

**Published:** 2026-02-22

**Authors:** Mingyue Zhou, Huijun Zhang

**Affiliations:** School of Nursing, Jinzhou Medical University, Jinzhou 121001, China

**Keywords:** frailty, empty-nest, older adults, trajectory, predictors

## Abstract

Background: Empty-nest older adults are considered a high-risk group for frailty due to constrained social support systems, yet the heterogeneity in their frailty progression remains poorly characterized. This study aimed to identify distinct frailty trajectory classes among Chinese empty-nest older adults and explore class-specific predictive factors. Methods: We analyzed eight years of data from the China Health and Retirement Longitudinal Study. The analysis included 1399 empty-nest older adults after eligibility screening. Frailty was assessed by the frailty index (FI). Growth Mixture Modeling was employed to identify FI trajectory classes, an linear, quadratic, and freely estimated forms were compared. Variable selection was performed via LASSO regression with bootstrap stability verification. Final predictors were analyzed using multinomial logistic regression. Results: A three-class quadratic model best represented the FI trajectories: “Low-increasing”, “High-fluctuating”, and “Elevated-stable”. Common risk factors included older age, rural residence, lower grip strength, death of children, and lower life satisfaction. The “High-fluctuating” trajectory was associated with poorer childhood health and smoking. The “Elevated-stable” trajectory was predicted by worklessness and by drinking. Physiological indicators showed no independent associations. Conclusions: Frailty among Chinese empty-nest older adults follows heterogeneous pathways shaped by life-course, socioeconomic, and psychophysiological factors. These results support the need for trajectory-specific screening, early risk detection, and tailored interventions for high-risk subgroups.

## 1. Introduction

It is estimated that by 2030, empty-nest households will account for more than half of all older adult households in China, with the number of empty-nest older adults exceeding 200 million [1]. This growing population faces distinct disadvantages in daily support and social connectedness. Compared to those living with children, they experience significant gaps in daily care, emergency support, and emotional comfort, with more fragile social support networks [2]. Frailty, a syndrome marked by diminished physiological reserve and multisystem dysfunction, is a key predictor of adverse outcomes like falls, disability, and hospitalization [3]. Therefore, precisely mapping frailty development in this population is crucial for establishing effective support systems and empowering healthier, more autonomous aging.

Frailty research has shifted from a “static risk” to a “dynamic process” paradigm. Early work identified risk factors at single time points [4,5], while later studies recognized multidimensional subtypes such as cognitive and psychological frailty [6,7,8]. Research has also expanded to include older adults with long-term conditions such as heart failure and lung cancer. These studies reveal interactions between frailty and disease progression and prognosis [9,10]. Frailty is now treated as a dynamic risk factor and focuses on its long-term impact on terminal health events like disability and mortality [4,11]. However, most studies have viewed frailty as homogeneous. They have yet to examine whether frailty follows heterogeneous trajectories in older subpopulations with unique sociopsychological characteristics.

Frailty is a dynamic and heterogeneous process rather than a static endpoint [12,13]. Latent class approaches such as Growth Mixture Modeling (GMM) have been applied to capture this intrinsic diversity. These methods have successfully identified representative patterns, including “stable” and “progressive” types [14,15]. Unlike traditional regression methods assuming homogeneity, GMM detects effectively data-driven subgroups, and utilizes all observed information through Full Information Maximum Likelihood estimation [16]. However, prior studies often conflated empty-nest older adults with the general older population, neglecting potential unique trajectories resulting from their absence of family support.

Based on this rationale, we utilized eight years of longitudinal data to explore latent class structures of frailty trajectories in Chinese empty-nest older adults. Key predictors were selected using LASSO regression, with stability confirmed through bootstrap resampling. This approach reveals intrinsic heterogeneity within this population and supports the construction of interpretable risk models. Our study provides national longitudinal evidence of heterogeneous frailty progression in empty-nest older adults, offering an evidence base for targeted community-level screening and intervention.

## 2. Materials and Methods

### 2.1. Study Design and Participants

Data were drawn from the China Health and Retirement Longitudinal Study (CHARLS), a nationally representative longitudinal cohort survey [17]. It collected information on demographic, social, economic, and health status from a sample of middle-aged and older residents in China. A multistage stratified probability-proportional-to-size sampling method was employed to ensure representativeness and minimize potential biases. The baseline sample covered 150 county-level districts across 28 provinces (autonomous regions, municipalities) in China. While CHARLS has conducted five survey waves (2011, 2013, 2015, 2018, 2020), this study utilized the first four waves to avoid potential confounding effects of the COVID-19 pandemic evident in the 2020 data.

**Figure 1:**
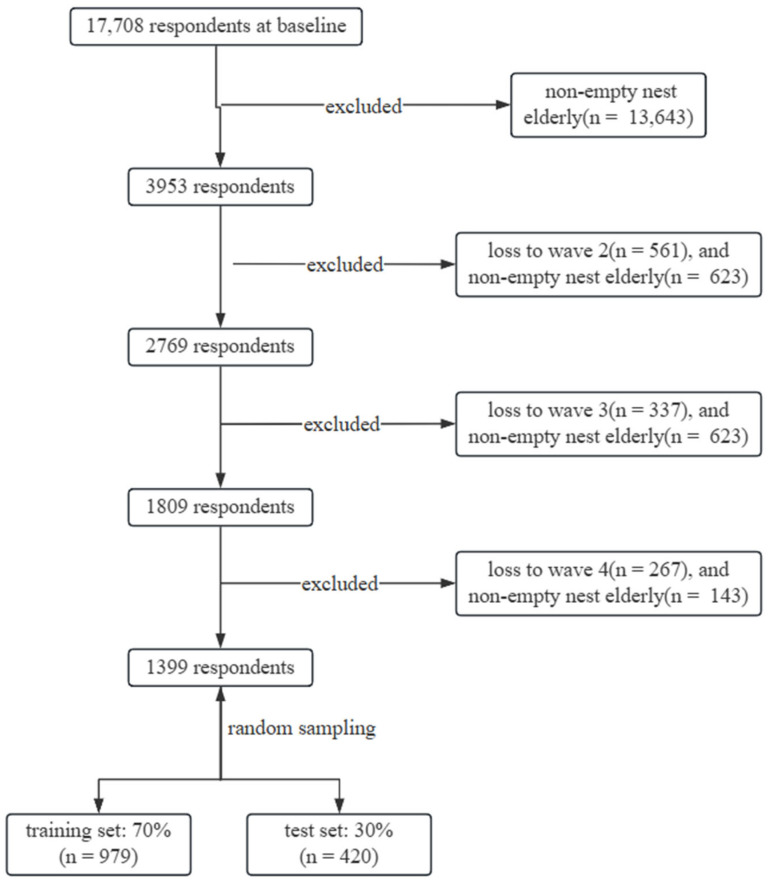
Flowchart of the study population selection process for empty-nest older adults. Note. Wave 1 refers to the 2011 survey, wave 2 to 2013, wave 3 to 2015, and wave 4 to 2018.

Empty-nest older adults were defined as individuals aged ≥ 60 years without co-residing children (including children-in-law), with children’s time away from home exceeding six months. From 3953 eligible participants at baseline, we excluded those lost to follow-up or who reverted to non-empty-nest status in any wave, resulting in 1399 participants for the final analysis (Figure 1).

### 2.2. Assessment of Frailty

Frailty was assessed using a Frailty Index (FI) based on the accumulation of age-related health deficits, following standard procedures [18,19]. A total of 32 items were selected from CHARLS covering comorbidities, physical function, disabilities, depression, and cognition (Table S1 in Supplementary Materials). All variables were dichotomized (0 = absent, 1 = present), except item 32, a continuous cognitive measure (0–1). Participants with >20% missing FI components were excluded [20]. The FI was calculated as the percentage of deficits present (range 0–100), with FI ≥ 25 defining frailty [21]. The index was computed separately across four CHARLS waves to track frailty progression through repeated measurements.

### 2.3. Covariates

A comprehensive set of potential confounding factors was selected to investigate how these variables influence FI trajectories among Chinese empty-nest older adults. The demographic component included age, sex, education, residence, and marital status. Physical measurements encompassed body mass index (BMI), grip strength, and waist circumference. Laboratory biomarkers comprised hemoglobin, C-reactive protein, glucose, glycated hemoglobin, total cholesterol, high-density lipoprotein cholesterol, low-density lipoprotein cholesterol, blood urea nitrogen, creatinine, and uric acid. The lifestyle section encompassed work status, living alone, children, social activities, smoking, drinking, and life satisfaction. The early life experiences section involved childhood health, motherly warmth, and death of children. Details are provided in Table S2 of the Supplementary Materials.

### 2.4. Statistical Analysis

Descriptive statistics presented continuous variables as means and standard deviations. These variables were compared using ANOVA. Categorical variables were expressed as frequencies and percentages. They were compared using chi-square or Fisher’s exact tests.

Frailty trajectories were analyzed by calculating each participant’s FI across four waves. GMM identified distinct trajectory classes, comparing linear, quadratic, and freely estimated forms. Model selection was guided by (1) Information Criteria, including the Akaike Information Criterion (AIC), Bayesian Information Criterion (BIC), and adjusted BIC (aBIC). Lower values indicate a better fit. (2) Likelihood Ratio Tests, including the Lo-Mendell-Rubin test (LMRT) and Bootstrap Likelihood Ratio Test (BLRT). A significant *p*-value supports a model with one additional trajectory class. (3) Entropy: Higher values indicate greater classification accuracy. Beyond these metrics, practical relevance and interpretability were also considered. Sequential analysis was performed by successively adding trajectory classes and comparing model metrics. All GMM analyses used 1000 random start values with 250 final iterations.

Variable selection was performed using LASSO regression to identify parsimonious predictors of frailty trajectory membership. The optimal penalty parameter (λ) was determined via 10-fold cross-validation following the “one standard error (1-SE)” rule, which favors a more parsimonious model while maintaining predictive accuracy. Clinically, this procedure identified which variables contribute robustly to distinguishing frailty trajectories after accounting for multicollinearity. Variables with non-zero coefficients in at least one trajectory class were retained as predictors. To evaluate the stability of this variable set, we conducted a bootstrap resampling procedure with 100 replicates. In each bootstrap sample, LASSO regression with 10-fold cross-validation (using the lambda.1se rule) was independently applied, ensuring that the selected predictors were not artifacts of sampling variability. Finally, the selected variables were included in a multinomial logistic regression model to predict trajectory membership.

Statistical analyses and graphing were performed using Mplus version 8.3 and R version 4.5.1. Missing data were handled using multiple imputation with the mice package. A significance level of 0.05 was adopted for all tests, and all *p*-values were two-sided.

## 3. Results

### 3.1. Characteristics of the Study Population

The demographic characteristics of 1399 participants and their FI scores across four waves are presented in Table S3 of the Supplementary Material. During the baseline survey, the average age was 67.45 years (SD = 5.83). The male-to-female ratio was approximately 1:1. Most participants had under secondary education, resided in rural areas, and were married.

### 3.2. Determination of the Optimal GMM

The model fitting results for the FI trajectories are detailed in Tables S4–S8 of the Supplementary Materials for each of the five imputed datasets. These tables compare the freely estimated, linear, and quadratic models. Model selection criteria prioritized minimizing AIC, BIC, and aBIC values while maximizing Entropy values. Both LMR and BLRT tests achieved statistical significance, balancing practical relevance and interpretability. Among the five datasets, three supported a three-class quadratic model, one supported a seven-class linear model, and another supported a six-class quadratic model. After comprehensive consideration, the three-class quadratic model was selected as the final model. The corresponding trajectory plots are shown in Figures S1–S5 of the Supplementary Materials.

Based on the model’s intercept, slope, and quadratic term parameters, three distinct FI trajectory patterns were identified (Table 1). Class 1 (68%) was named “Low-increasing”, with a baseline FI mean of 10.29 ± 6.92, indicating a non-frail state. Although this group showed a gradual upward trend throughout the observation period, its FI consistently remained below the frailty threshold, reaching 15.58 ± 8.49 at the endpoint. Class 2 (21%) was termed “High-fluctuating”, with baseline levels already reaching frailty status (26.93 ± 13.76). This trajectory featured a brief mid-term improvement (Wave 2: 20.63 ± 10.71) followed by a sharp deterioration in health status, reaching 43.48 ± 15.06 by Wave 4. Class 3 (11%) was designated “Elevated-stable”, exhibiting the highest baseline FI (27.97 ± 16.98). This group experienced rapid health deterioration in the early phase, after which frailty levels plateaued at a high plateau phase, ultimately stabilizing at 45.86 ± 16.63. Additionally, comparisons of baseline characteristics among frailty trajectory groups are presented in Table S3 of the Supplementary Material.

### 3.3. Variable Selection

**Figure 2:**
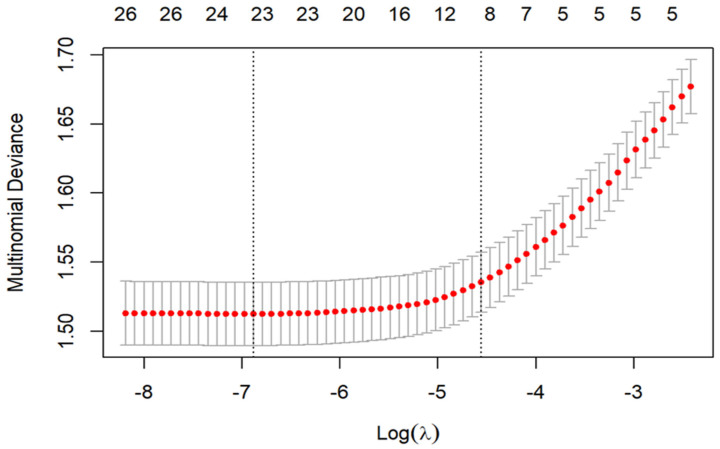
Lasso cross-validation curve on FI trajectories of empty-nest older adults. Note. The top axis of the plot indicates the number of key predictor variables corresponding to different log(λ) values. The left vertical dashed line represents the log(λ) value at which the minimal deviance was achieved (lambda.min). The right vertical dashed line corresponds to the log(λ) value that is one standard error away from the minimal deviance (lambda.lse).

**Figure 3:**
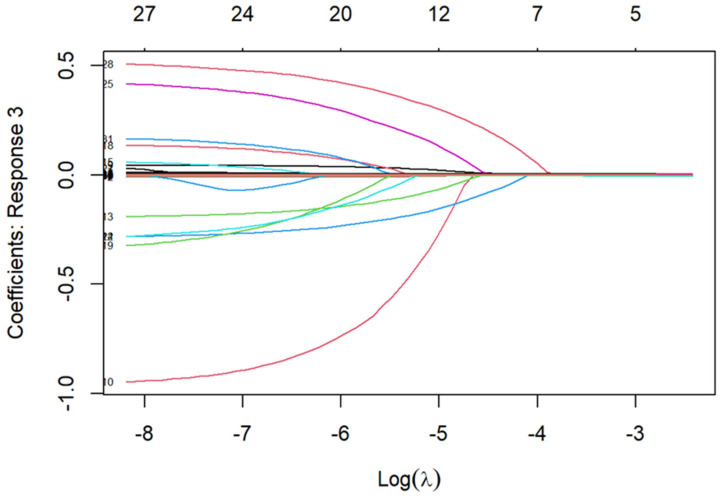
LASSO coefficients for 18 variables.

LASSO regression on 28 candidate predictors identified 18 variables, including age, education, residence, BMI, grip strength, waist circumference, glycated hemoglobin, total cholesterol, glucose, blood urea nitrogen, creatinine, uric acid, work status, smoking, drinking, childhood health, death of children, and life satisfaction (see Figure 2 and Figure 3; coefficient table in Table S9 of the Supplementary Materials). Figure 2 visualized the trade-off between model complexity and prediction error across a range of λ values. Figure 3 displayed the coefficients of candidate predictors across λ values, with non-zero coefficients at the selected λ retained as predictors. Bootstrap resampling consistently selected the same 18-variable set, confirming its robustness. A heatmap of selection frequency across bootstrap samples is provided in Figure S6 of the Supplementary Materials.

### 3.4. Determinants of Trajectory Membership

Multinomial logistic regression, using the “Low-increasing” trajectory as the reference, identified distinct predictors for the two high-risk trajectories (Table 2). Older age was a universal risk factor, while greater grip strength and higher life satisfaction were universally protective. Trajectory-specific analysis revealed that poorer childhood health, smoking, rural residence, and death of children significantly predicted the “High-fluctuating” trajectory, whereas worklessness, drinking, death of children, and rural residence were associated with the “Elevated-stable” trajectory. Notably, experience of child loss was a common risk factor across both high-risk trajectories, and rural residence also increased the likelihood of both. In contrast, physiological biomarkers such as glycated hemoglobin and total cholesterol showed no independent associations with trajectory membership.

## 4. Discussion

Due to the lack of daily care and support from their children, empty-nest older adults face unique healthy aging challenges. Using eight-year CHARLS data, this study identified three distinct FI trajectories via GMM and their key predictors using LASSO regression with bootstrap validation. These findings provide empirical evidence for refined health management strategies for this vulnerable population.

GMM analysis revealed three distinct FI trajectories among empty-nest older adults, designated as “Low-increasing,” “High-fluctuating,” and “Elevated-stable.” Previous studies also commonly report a three-class structure dominated by a low-stable subgroup [15,22]. This pattern may be driven by gradual physiological decline alongside manageable long-term conditions. The stable non-frail status is potentially attributable to stronger physiological reserve or healthier baseline status [23]. However, despite the similarity in trajectory shapes, the frailty burden was higher in our empty-nest sample than levels typically reported in general aging cohorts. This disparity highlights the accumulated disadvantages faced by empty-nest older adults in terms of social support, psychological resources, and access to care. Most previous studies have reported relatively static or unidirectional patterns [24]. In contrast, our findings show varied and non-uniform pathways, suggesting that frailty is shaped by multiple interacting factors. This observation is consistent with the limited existing evidence for nonlinear frailty progression, such as that reported by Gajic-Veljanoski et al. [25].

The “High-fluctuating” trajectory showed an initial improvement in frailty followed by a sharp decline. This pattern may reflect temporary health gains during mid-follow-up, such as recovery from an acute illness or improvements in living conditions. These gains may have been followed by accelerated age-related decline. Although individual variability exists, this non-monotonic trend was consistently observed across most individuals in this class. Further studies are needed to confirm this finding and explore its underlying mechanisms.

Furthermore, some studies have identified different class numbers, such as two [26], four [22,27], or five [3]. These discrepancies may arise from variations in frailty measurement indicators, follow-up duration and intervals, or sample characteristics [28,29]. Differences in healthcare, socioeconomic conditions, and culture across countries or regions further contribute to variations. For instance, Southern European populations exhibit steeper FI progression than Northern Europeans, and regular participation in cultural activities is associated with slower frailty progression [30,31].

Given the heterogeneity in frailty progression, identifying trajectory predictors can guide stratification of empty-nest older adults. Handgrip strength and life satisfaction demonstrated protective effects across trajectories. These factors provide dual physiological and psychological support for maintaining the relatively stable “Low-increasing” trajectory [32,33]. In contrast, the risk effect of advanced age, as an irreversible physiological process, was also confirmed in this study.

Rural residence and child loss were identified as shared key risk contexts for the two high-risk trajectories. Rural residence is associated with limited access to medical and social services and relative socioeconomic resource deprivation, which constitute a foundational environment for health vulnerability [34,35]. Child loss, as a major psychological trauma and life event, not only causes profound psychological distress but may also disrupt core family support networks and economic resources [36].

Against this shared backdrop, the two high-risk trajectories exhibited distinct risk profiles. The “High-fluctuating” trajectory was characterized by the additional factors of smoking and poorer childhood health. One possible explanation is that poorer childhood health may impair the development of physiological reserve. This early-life disadvantage may accumulate over the life course and increase vulnerability to later-life stressors [37,38]. This group experiences mid-term fluctuations before health reserves deplete, eventually leading to rapid decompensation. In contrast, the “Elevated-stable” trajectory was primarily driven by accumulated socioeconomic disadvantages and behavioral risks. Its key features included worklessness (unemployment, retirement, or never employed) and alcohol consumption. Worklessness implies income instability and loss of social roles, while rural residence further constrains access to resources [39,40]. Within this structural context, alcohol consumption may serve as a coping strategy while further accelerating health deterioration. The experience of child loss demonstrated a particularly significant risk effect in this trajectory. It acted synergistically with existing disadvantages, such as worklessness and alcohol use. Collectively, these factors rapidly depleted health reserves, leading individuals to a state of low-level but high-vulnerability plateau rather than genuine stability.

Notably, physiological indicators showed no independent associations when multidimensional factors were considered. This finding suggests that psychosocial and behavioral factors may be more directly related to frailty trajectory membership than baseline physiological biomarkers among empty-nest older adults. These biomarkers may reflect long-term health deficits already captured by the FI, or their effects may be mediated through social and behavioral pathways identified in this study. Further research is needed to clarify the temporal and causal relationships among these domains.

Several limitations should be considered. First, frailty was assessed using the FI derived from the CHARLS database. This index is based on self-reported items and physical measurements rather than direct clinical evaluations, which may introduce measurement bias. Second, excluding participants who did not complete all follow-up surveys may have led to selection bias toward healthier survivors. This could result in underestimation of frailty severity and overrepresentation of robust individuals in high-risk trajectories. Third, although CHARLS is a widely used database, measurement error cannot be completely ruled out. The generalizability of our findings also requires validation in other populations and settings. Fourth, despite adjusting for multiple covariates, unmeasured confounding factors—such as genetic predispositions or detailed psychosocial stressors—may remain. Finally, because our analysis used data only up to 2018, the findings may not reflect post-pandemic changes in risk factors, social support, or healthcare access for empty-nest older adults. This may limit the applicability of our results to the post-pandemic context.

## 5. Conclusions

Through long-term tracking of empty-nest older adults, this study established three FI trajectories: “Low-increasing,” “High-fluctuating,” and “Elevated-stable.” It also identified specific predictors for each trajectory, providing key evidence for early warning and targeted care. Future efforts can focus on creating tailored prevention strategies for each trajectory type, building effective screening and stepped-care systems, and implementing systemic interventions to alter the course of frailty. In addition, longitudinal studies that follow older adults from the pre-empty-nest phase through the transition period are needed to identify critical windows during which social vulnerability progresses into physical frailty. Future research can also examine the association between the identified frailty trajectories and hard clinical outcomes, such as mortality, disability, and hospitalization, to further establish their prognostic relevance. These steps will provide concrete support for healthy aging in this population.

## Figures and Tables

**Table 1:** Parameter estimates of the three-class quadratic model.

Classes	N (%)	Intercept	P	Slope	P	Quadratic	P
Class 1	948 (0.68)	10.045	<0.001	0.688	0.056	0.370	0.002
Class 2	294 (0.21)	25.675	<0.001	−10.861	<0.001	5.369	<0.001
Class 3	157 (0.11)	26.331	<0.001	15.193	<0.001	−3.160	0.002

Note. Class 1, Low-increasing; Class 2, High-fluctuating; Class 3, Elevated-stable.

**Table 2:** Multinomial analysis of the three-class quadratic model.

Variables	OR (95% CI) [“Low-Increasing” as Reference]	
	High-Fluctuating	Elevated-Stable
Age	1.06 (1.03–1.09) ***	1.07 (1.03–1.10) ***
Education (Lower secondary as reference)		
Other	0.65 (0.32–1.34)	0.51 (0.17–1.56)
Residence (Urban as reference)		
Rural	1.95 (1.27–3.00) **	2.34 (1.37–3.98) **
BMI (18.5 to 22.9 as reference)		
0 to 18.4	1.37 (0.82–2.31)	1.09 (0.52–2.25)
23 to 24.9	0.84 (0.55–1.30)	0.73 (0.39–1.36)
25 to 100	1.41 (0.90–2.19)	1.46 (0.80–2.66)
Grip strength	0.97 (0.95–0.98) ***	0.95 (0.93–0.98) ***
Waist	1.01 (0.99–1.03)	1.01 (0.99–1.03)
HbA1c	1.06 (0.86–1.32)	1.11 (0.79–1.55)
TC	1.00 (1.00–1.01)	1.00 (1.00–1.01)
Glucose	1.00 (1.00–1.01)	1.00 (0.99–1.01)
BUN	1.00 (0.96–1.05)	1.05 (0.99–1.10)
Creatinine	0.88 (0.34–2.29)	0.31 (0.09–1.10)
UA	0.88 (0.76–1.02)	0.97 (0.80–1.17)
Work status (Non-agricultural as reference)		
Agricultural	1.14 (0.67–1.92)	0.97 (0.46–2.03)
Workless	1.55 (0.89–2.68)	2.23 (1.08–4.64) *
Smoking (No as reference)		
Yes	1.51 (1.08–2.12) *	1.46 (0.93–2.27)
Drinking (No as reference)		
Yes	1.14 (0.83–1.58)	1.66 (1.09–2.53) *
Childhood health	1.17 (1.01–1.35) *	1.09 (0.90–1.33)
Death of children (No as reference)		
Yes	1.98 (1.37–2.86) ***	1.63 (1.00–2.67) *
Life satisfaction	0.73 (0.58–0.92) **	0.56 (0.41–0.75) ***

Note. BMI, body mass index; HbA1c, glycated hemoglobin; TC, total cholesterol; BUN, blood urea nitrogen; UA, uric acid; *, *p*  <  0.05; **, *p*  <  0.01; ***, *p*  <  0.001; OR, odds ratio; CI, confidence intervals.

## Data Availability

The datasets analyzed during the current study are available in the CHARLS repository: http://charls.pku.edu.cn/ (accessed on 4 March 2025).

## Supplementary Materials

The following supporting information can be downloaded at: https://www.mdpi.com/article/10.3390/healthcare14040537/s1, Figure S1: Trajectories of FI for empty-nest older adults in three-class quadratic growth mixture model in the first imputation dataset; Figure S2: Trajectories of FI for empty-nest older adults in six-class quadratic growth mixture model in the second imputation dataset; Figure S3: Trajectories of FI for empty-nest older adults in seven-class linear growth mixture model in the third imputation dataset; Figure S4: Trajectories of FI for empty-nest older adults in three-class quadratic growth mixture model in the fourth imputation dataset; Figure S5: Trajectories of FI for empty-nest older adults in three-class quadratic growth mixture model in the fifth imputation dataset; Figure S6: The heatmap of selection frequency across bootstrap samples; Table S1: The 32 items used to construct the FI; Table S2: Covariates: measuring methods, variable type, and units/categories; Table S3: Participant characteristics by FI from Wave 1 to Wave 4; Table S4: Model selection criteria for the GMM analysis in the first imputation dataset; Table S5: Model selection criteria for the GMM analysis in the second imputation dataset; Table S6: Model selection criteria for the GMM analysis in the third imputation dataset; Table S7: Model selection criteria for the GMM analysis in the fourth imputation dataset; Table S8: Model selection criteria for the GMM analysis in the fifth imputation dataset; Table S9: Variables and coefficients selected by LASSO.

## Abbreviations

GMM	Growth Mixture Modeling
CHARLS	the China Health and Retirement Longitudinal Study
FI	Frailty Index
BMI	body mass index
CRP	C-reactive protein
HbA1c	glycated hemoglobin
TC	total cholesterol
HDL-C	high-density lipoprotein cholesterol
LDL-C	low-density lipoprotein cholesterol
BUN	blood urea nitrogen
UA	uric acid
AIC	Akaike Information Criterion
BIC	Bayesian Information Criterion
aBIC	adjusted BIC
LMRT	Lo-Mendell-Rubin test
BLRT	Bootstrap Likelihood Ratio Test
OR	odds ratio
CI	confidence intervals
