# Supplementary material for: Frailty Trajectories and Their Predictors in Chinese Empty-Nest Older Adults: An 8-Year Longitudinal Study
Source: Healthcare (Basel). 2026 Feb 22;14(4):537. doi: 10.3390/healthcare14040537 (PMC12940645; doi:10.3390/healthcare14040537)
Supplement: Supplementary file 1 [file healthcare-14-00537-s001.zip › healthcare-4098227-supplementary.pdf]

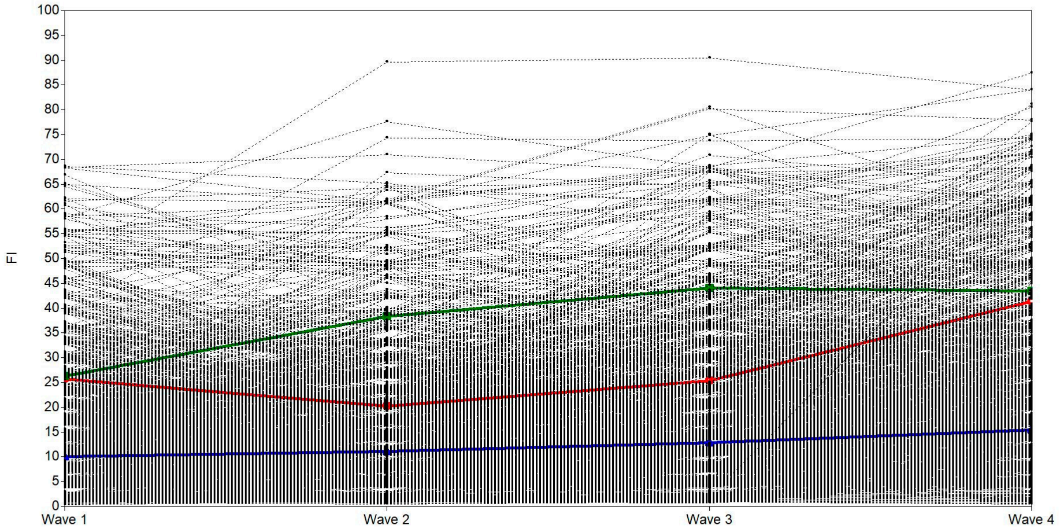

Figure S1 Trajectories of FI for empty-nest older adults in three-class quadratic growth mixture model in the first imputation dataset

Note: Wave 1 refers to the 2011 survey; Wave 2 refers to the 2013 survey; Wave 3 refers to the 2015 survey; Wave 4 refers to the 2018 survey.

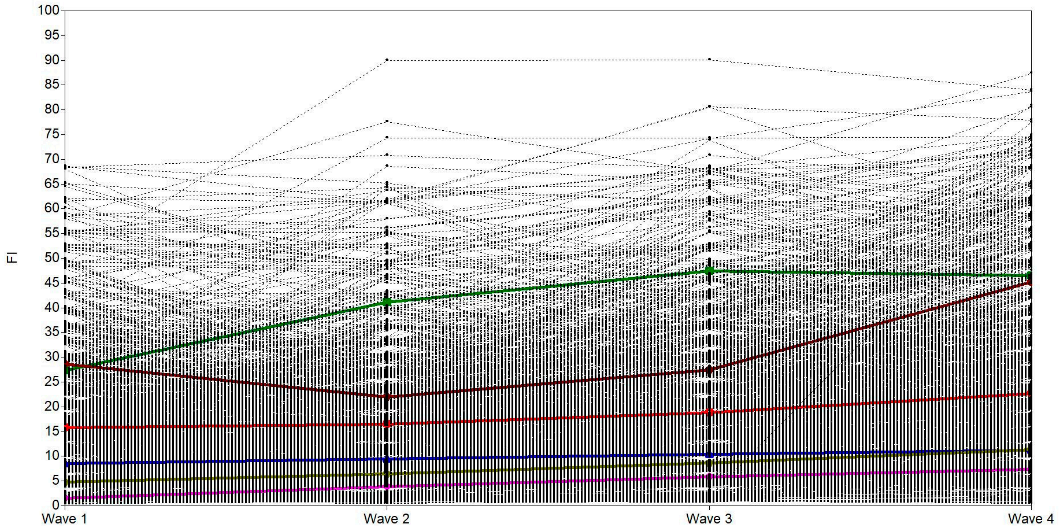

Figure S2. Trajectories of FI for empty-nest older adults in six-class quadratic growth mixture model in the second imputation dataset

Note: Wave 1 refers to the 2011 survey; Wave 2 refers to the 2013 survey; Wave 3 refers to the 2015 survey; Wave 4 refers to the 2018 survey.

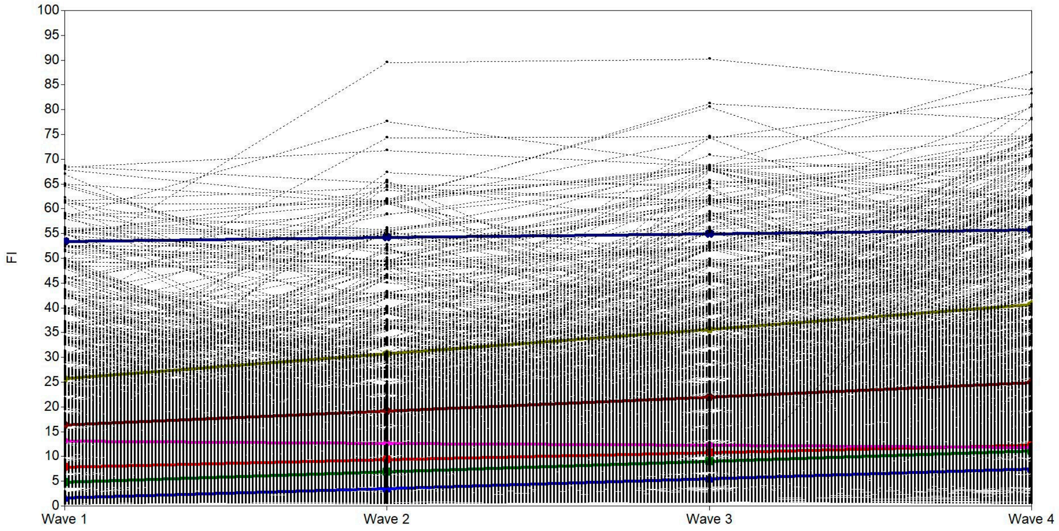

Figure S3. Trajectories of FI for empty-nest older adults in seven-class linear growth mixture model in the third imputation dataset

Note: Wave 1 refers to the 2011 survey; Wave 2 refers to the 2013 survey; Wave 3 refers to the 2015 survey; Wave 4 refers to the 2018 survey.

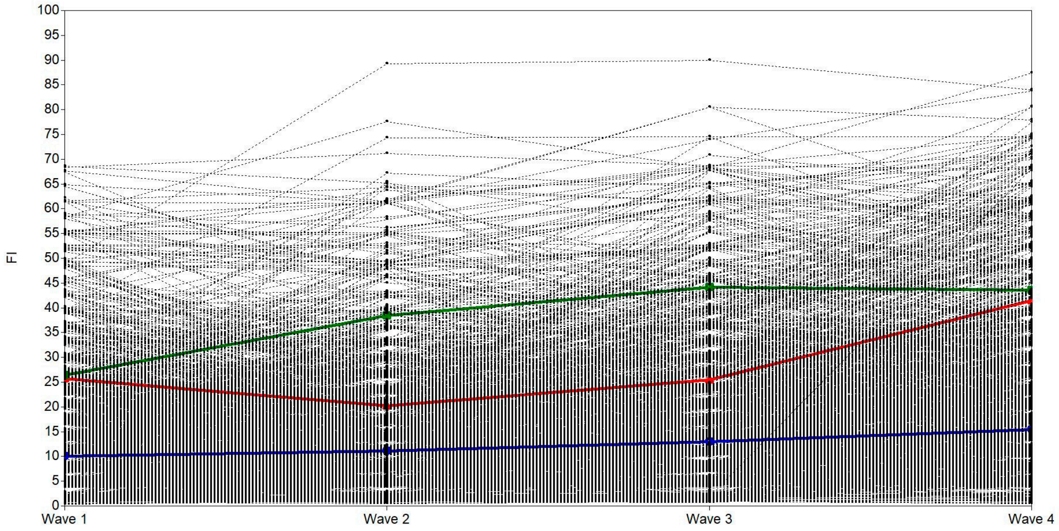

Figure S4. Trajectories of FI for empty-nest older adults in three-class quadratic growth mixture model in the fourth imputation dataset

Note: Wave 1 refers to the 2011 survey; Wave 2 refers to the 2013 survey; Wave 3 refers to the 2015 survey; Wave 4 refers to the 2018 survey.

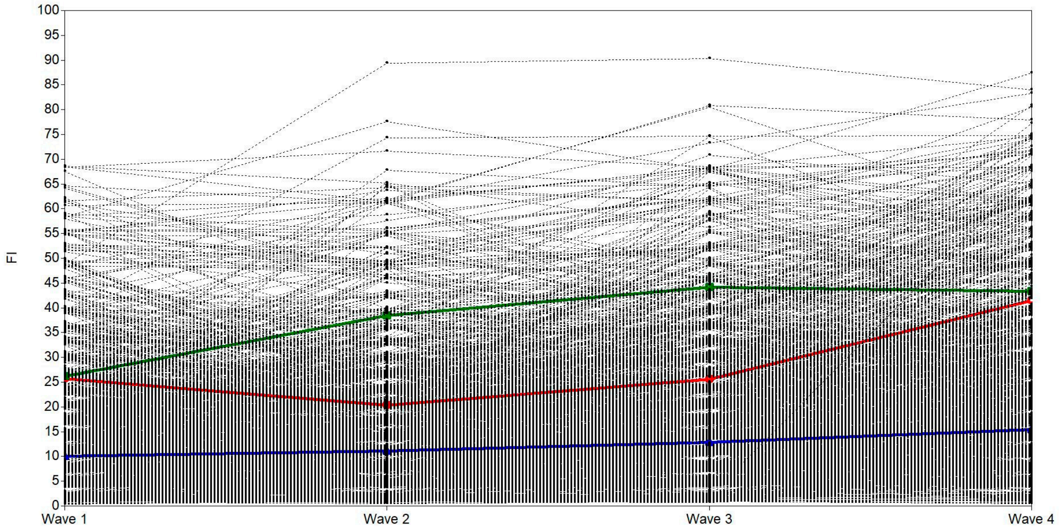

Figure S5. Trajectories of FI for empty-nest older adults in three-class quadratic growth mixture model in the fifth imputation dataset

Note: Wave 1 refers to the 2011 survey; Wave 2 refers to the 2013 survey; Wave 3 refers to the 2015 survey; Wave 4 refers to the 2018 survey.

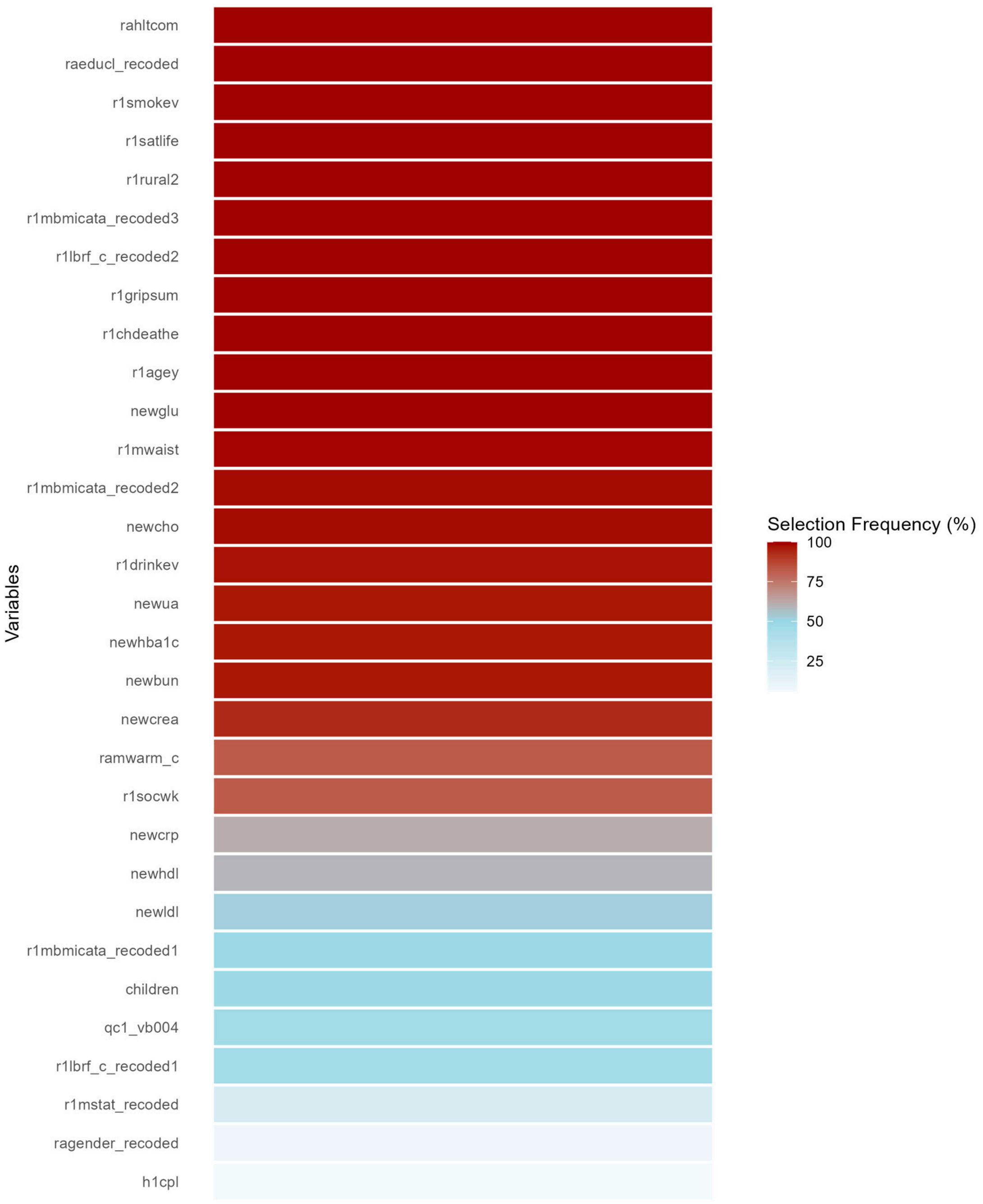

Figure S6. The heatmap of selection frequency across bootstrap samples

Table S1. The 32 items used to construct the FI.

Individual components	Description of the items	Criteria
Self-reported diseases	Physician diagnosed hypertension	Yes = 1, No = 0
	Physician diagnosed diabetes	Yes = 1, No = 0
	Physician diagnosed heart disease	Yes = 1, No = 0
	Physician diagnosed stroke	Yes = 1, No = 0
	Physician diagnosed cancer	Yes = 1, No = 0
	Physician diagnosed arthritis	Yes = 1, No = 0
	Physician diagnosed chronic lung disease	Yes = 1, No = 0
	Physician diagnosed asthma	Yes = 1, No = 0
	Physician diagnosed any emotional, nervous, or psychiatric problems	Yes = 1, No = 0
	Physician diagnosed memory-related disease	Yes = 1, No = 0
Self-reported vision and hearing issues	Vision problems	Yes = 1, No = 0
	Hearing problems	Yes = 1, No = 0
Self-reported general and mental health assessment	Self-reported general health status	Very poor or poor = 1, very good, good or fair = 0
	Depression: CESD-10 items	CESD-10 >10 =1, ≤10 =0
	Cognition: (memory score + orientation score) / 14	from 0 to 1
Activities of daily living (ADLs)	Some difficulty dressing	Yes = 1, No = 0
	Some difficulty bathing or showering	Yes = 1, No = 0
	Some difficulty eating	Yes = 1, No = 0
	Some difficulty getting in and out of bed	Yes = 1, No = 0

	Some difficulty using the toilet	Yes = 1, No = 0
Instrumental activities of daily living (IADLs)	Some difficulty managing money	Yes = 1, No = 0
	Some difficulty taking medications	Yes = 1, No = 0
	Some difficulty shopping for groceries	Yes = 1, No = 0
	Some difficulty preparing meals	Yes = 1, No = 0
	Some difficulty cleaning house	Yes = 1, No = 0
Mobility status	Some difficulty walking 100M	Yes = 1, No = 0
	Some difficulty getting up from a chair after sitting for long periods	Yes = 1, No = 0
	Some difficulty climbing several flights of stairs without resting	Yes = 1, No = 0
	Some difficulty stooping, kneeling, or crouching	Yes = 1, No = 0
	Some difficulty lifting or carrying weights over 10 jins (approximately 5 kilograms)	Yes = 1, No = 0
	Some difficulty picking up a coin from the table	Yes = 1, No = 0
	Some difficulty reaching arms above shoulder level	Yes = 1, No = 0

Heart disease indicates angina, coronary heart disease, congestive heart failure, or other heart problems.

Memory-related disease indicates Alzheimer's disease or dementia, organic brain senility, or other serious memory impairment.

Depression is evaluated using the Center for Epidemiologic Studies Depression Scale (CESD). In the CHARLS, CESD-10 is used, and the total score ranges from 0 to 30. A higher score indicates more severe depressive symptoms.

The memory score is the average of words that are not recalled in the immediate and delayed word recall tasks. The memory score ranges from 0 to 10. The orientation test comprises four questions about the day of the week, the month, the date of the month, and the year. One point is given for each wrong answer, and the range is from 0 to 4.

Table S2. Covariates: measuring methods, variable type, and units/categories.

Covariates	Measuring methods	Variable type	Units/categories
Age	Actual age.	Continuous	years
Sex	Biological sex.	Categorical	0 “man”; 1 “woman”
Education	Highest educational attainment. Respondents were categorized into two groups: lower secondary (less than lower secondary) and others (upper secondary education or vocational training and tertiary education).	Categorical	0 “lower secondary”; 1 “other”
Residence	Type of residential location.	Categorical	0 “urban”; 1 “rural”
Marital status	Current state.	Categorical	0 “Married”; 1 “Others”
BMI	Body mass index: Weight divided by the square of the height. The BMI category according to the WHO recommendation for the Asian and South Asian population was: underweight, less than 18.5; normal weight, from 18.5 to 22.9; overweight, from 23 to 24.9; and obesity, from 25 to 100.	Categorical	0 “0 to 18.5”; 1 “18.5 to 22.9”; 2 “23 to 24.9”; 3 “25 to 100”
Grip strength	The maximum measurement of the dominant hand. For respondents with equally dominant hands, the greater value between two hands was used.	Continuous	kg
Waist	Measured waist.	Continuous	cm
Hemoglobin	Blood sample testing.	Continuous	g/dL
CRP	Blood sample testing.	Continuous	mg/L
HbA1c	Blood sample testing.	Continuous	%
TC	Blood sample testing.	Continuous	mg/dL
HDL-C	Blood sample testing.	Continuous	mg/dL
LDL-C	Blood sample testing.	Continuous	mg/dL

Glucose	Blood sample testing.	Continuous	mg/dL
BUN	Blood sample testing.	Continuous	mg/dL
Creatinine	Blood sample testing.	Continuous	mg/dL
UA	Blood sample testing.	Continuous	mg/dL
Work status	It was categorized into agricultural, non-agricultural, and workless. Agricultural included employees and self-employed. Non-agricultural included employees, self-employed, and family businesses. Workless included the unemployed, the retired, and those who had never worked.	Categorical	0 “agricultural”; 1 “non-agricultural”; 2 “workless”
Live alone	Current state.	Categorical	0 “No”; 1 “Yes”
Children.	Current state.	Categorical	0 “No”; 1 “Yes”
Social activities	Current state.	Categorical	0 “No”; 1 “Yes”
Smoking	Ever smoked at any time.	Categorical	0 “No”; 1 “Yes”
Drinking	Ever drank alcohol at any time.	Categorical	0 “No”; 1 “Yes”
Childhood health	Health condition compared to other children of same age before age 16. The poorer the child’s health, the higher the score.	Continuous	NA
Mother warmth	A measure of mean maternal warmth. It was based on three components: the effort the female guardian put into watching them, the love and affection the female guardian gave them, and the self-rated relationship with their female guardian while respondents were growing up. The maternal affection increased with the score.	Continuous	NA
Death of children	Had experienced the death of children.	Categorical	0 “No”; 1 “Yes”
Life satisfaction	Respondent’s level of life satisfaction. Responses were recorded on a 5-point Likert scale so that higher scores indicated greater satisfaction.	Continuous	NA

Note. BMI, body mass index; CRP, C-reactive protein; HbA1c, glycated hemoglobin; TC, total cholesterol; HDL-C, high-density lipoprotein cholesterol; LDL-C, low-density lipoprotein cholesterol; BUN, blood urea nitrogen; UA, uric acid; NA, not applicable.

Table S3. Participant characteristics by FI from Wave 1 to Wave 4.

Variables	N (%) / (Mean ± SD)				P
	N = 1399	Class 1(N = 948)	Class 2 (N = 294)	Class 3 (N = 157)	
Age	67.45 ± 5.83	66.67 ± 5.44	68.89 ± 6.17	69.43 ± 6.47	< 0.001
Sex					0.002
Man	724 (51.8)	521 (55.0)	135 (45.8)	68 (43.4)	
Woman	675 (48.2)	427 (45.0)	159 (54.2)	89 (56.6)	
Education					0.001
Lower secondary	1299 (92.9)	864 (91.1)	283 (96.1)	152 (97.1)	
Other	100 (7.1)	84 (8.9)	11 (3.9)	5 (2.9)	
Residence					< 0.001
Urban	358 (25.6)	278 (29.3)	53 (18.2)	27 (16.9)	
Rural	1041 (74.4)	670 (70.7)	241 (81.8)	130 (83.1)	
Marital status					0.210
Married	1192 (85.2)	818 (86.3)	245 (83.1)	129 (82.2)	
Others	207 (14.8)	130 (13.7)	49 (16.9)	28 (17.8)	
BMI					0.025
0 to 18.4	115 (8.2)	67 (7.0)	32 (11.1)	16 (10.1)	
18.5 to 22.9	587 (42.0)	404 (42.6)	118 (40.3)	65 (41.1)	
23 to 24.9	276 (19.7)	207 (21.9)	47 (16.0)	22 (14.1)	
25 to 100	421 (30.1)	270 (28.5)	97 (32.6)	54 (34.6)	
Grip strength	29.82 ± 10.00	31.35 ± 9.73	27.04 ± 9.96	25.82 ± 9.46	< 0.001
Waist	85.17 ± 13.15	84.74 ± 12.60	85.86 ± 14.10	86.47 ± 14.40	0.197
Hemoglobin	14.31 ± 2.21	14.4 ± 2.27	14.13 ± 1.99	14.13 ± 2.14	0.116
CRP	2.83 ± 7.79	2.70 ± 8.13	3.02 ± 6.97	3.28 ± 7.12	0.586
HbA1c	5.32 ± 0.88	5.27 ± 0.77	5.44 ± 1.09	5.40 ± 1.03	0.011
TC	197.39 ± 40.42	195.58 ± 38.14	202.54 ± 45.77	198.73 ± 42.15	0.049
HDL-C	52.49 ± 16.46	52.42 ± 16.31	53.22 ± 16.46	51.53 ± 17.33	0.513
LDL-C	120.51 ± 36.87	119.42 ± 35.37	124.4 ± 41.54	119.82 ± 36.15	0.162

Glucose	111.70 ± 37.26	109.77 ± 32.82	116.91 ± 48.03	113.59 ± 38.33	0.012
BUN	16.51 ± 4.60	16.45 ± 4.46	16.39 ± 4.72	17.12 ± 5.10	0.225
Creatinine	0.81 ± 0.21	0.82 ± 0.22	0.79 ± 0.20	0.78 ± 0.19	0.022
UA	4.59 ± 1.23	4.65 ± 1.25	4.45 ± 1.17	4.55 ± 1.19	0.068
Work status					< 0.001
Agricultural	585 (41.8)	414 (43.7)	121 (41.1)	50 (31.7)	
Non-agricultural	164 (11.7)	130 (13.7)	23 (8.0)	11 (6.6)	
Workless	650 (46.5)	404 (42.6)	150 (50.9)	96 (61.7)	
Live alone					0.203
No	224 (16.0)	141 (14.8)	53 (18.2)	30 (19.1)	
Yes	1175 (84.0)	807 (85.2)	241 (81.8)	127 (80.9)	
Children					0.618
No	64 (4.6)	44 (4.6)	11 (3.7)	9 (5.7)	
Yes	1335 (95.4)	904 (95.4)	283 (96.3)	148 (94.3)	
Social activities					0.396
No	722 (51.6)	478 (50.4)	160 (54.5)	84 (53.8)	
Yes	677 (48.4)	470 (49.6)	134 (45.5)	73 (46.2)	
Smoking					0.945
No	802 (57.3)	541 (57.1)	170 (57.6)	91 (58.2)	
Yes	597 (42.7)	407 (42.9)	124 (42.4)	66 (41.8)	
Drinking					0.501
No	800 (57.2)	538 (56.7)	176 (60.0)	86 (54.8)	
Yes	599 (42.8)	410 (43.3)	118 (40.0)	71 (45.2)	
Childhood health	2.67 ± 0.98	2.63 ± 0.97	2.79 ± 1.00	2.74 ± 1.02	0.041
Mother warmth	1.84 ± 0.74	1.83 ± 0.73	1.90 ± 0.79	1.80 ± 0.70	0.271
Death of children					< 0.001
No	1196 (85.5)	844 (89.0)	228 (77.3)	124 (79.2)	
Yes	203 (14.5)	104 (11.0)	66 (22.7)	33 (20.8)	
Life satisfaction	3.14 ± 0.68	3.20 ± 0.64	3.05 ± 0.68	2.93 ± 0.83	< 0.001

Note. BMI, body mass index; CRP, C-reactive protein; HbA1c, glycated hemoglobin; TC, total cholesterol; HDL-C, high-density lipoprotein cholesterol; LDL-C, low-density lipoprotein cholesterol; BUN, blood urea nitrogen; UA, uric acid.

Table S4. Model selection criteria for the GMM analysis in the first imputation dataset.

Class	LL	AIC	BIC	aBIC	Entropy	LMR(P)	BLRT(P)
Free Estimation							
1C	-21198.563	42419.127	42476.805	42441.862	-	-	-
2C	-20678.086	41394.172	41493.798	41433.443	0.695	<0.001	<0.001
3C	-20494.738	41043.476	41185.050	41099.282	0.641	0.0039	<0.001
**4C**	**-20355.432**	**40780.865**	**40964.388**	**40853.206**	**0.708**	**<0.001**	**<0.001**
5C	-20241.315	40568.631	40794.102	40657.507	0.717	0.1114	<0.001
Linear Estimation							
1C	-21251.139	42520.278	42567.470	42538.880	-	-	-
2C	-20860.166	41750.333	41828.985	41781.336	0.617	<0.001	<0.001
3C	-20746.950	41535.900	41646.014	41579.304	0.650	<0.001	<0.001
4C	-20703.570	41461.139	41602.714	41516.945	0.708	0.0182	<0.001
5C	-20672.337	41410.674	41583.710	41478.881	0.715	0.0032	<0.001
6C	-20574.236	41226.471	41430.968	41307.080	0.778	0.0279	<0.001
**7C**	**-20555.753**	**41201.506**	**41437.464**	**41294.516**	**0.790**	**0.0338**	**<0.001**
8C	-20482.642	41067.285	41334.704	41172.696	0.822	0.5442	0.1111
Quadratic Estimation							
1C	-21187.409	42400.818	42468.984	42427.688	-	-	-
2C	-20759.168	41560.335	41670.449	41603.740	0.676	<0.001	<0.001
**3C**	**-20612.067**	**41282.134**	**41434.196**	**41342.074**	**0.781**	**0.0061**	**<0.001**
4C	-20490.645	41055.291	41249.301	41131.765	0.739	<0.001	<0.001
5C	-20411.678	40913.356	41149.314	41006.366	0.766	0.0034	<0.001
6C	-20334.748	40775.497	41053.403	40885.042	0.788	0.3651	<0.001

Note: LL, Log Likelihood; AIC, Akaike Information Criteria; BIC, Bayesian Information Criterion; aBIC, sample size-adjusted BIC; LMR(P), the p-value of the Lo-Mendell-Rubin test; BLRT(P), the p-value of the Bootstrapped likelihood ratio test; 1C, one-class; 2C, two-class; 3C, three-class; 4C, four-class; 5C, five-class; 6C, six-class; 7C, seven-class; 8C, eight-class.

Table S5. Model selection criteria for the GMM analysis in the second imputation dataset.

Class	LL	AIC	BIC	aBIC	Entropy	LMR(P)	BLRT(P)
Free Estimation							
1C	-21201.323	42424.646	42482.325	42447.382	-	-	-
2C	-20680.626	41399.252	41498.879	41438.523	0.699	0.0003	<0.001
3C	-20496.831	41047.662	41189.237	41103.468	0.673	0.0544	<0.001
4C	-20364.222	40798.444	40981.967	40870.785	0.708	0.0001	<0.001
**5C**	**-20245.753**	**40577.506**	**40802.977**	**40666.382**	**0.721**	**0.0016**	**<0.001**
6C	-20159.659	40421.317	40688.736	40526.728	0.713	0.0658	<0.001
Linear Estimation							
1C	-21253.558	42525.116	42572.308	42543.718	-	-	-
2C	-20861.270	41752.541	41831.193	41783.544	0.618	<0.001	<0.001
3C	-20748.562	41539.125	41649.239	41582.529	0.649	<0.001	<0.001
4C	-20706.281	41466.561	41608.136	41522.367	0.708	0.0500	<0.001
**5C**	**-20678.080**	**41422.159**	**41595.195**	**41490.366**	**0.716**	**0.0034**	**<0.001**
6C	-20578.886	41235.773	41440.270	41316.382	0.795	0.2719	<0.001
Quadratic Estimation							
1C	-21190.097	42406.194	42474.360	42433.064	-	-	-
2C	-20761.120	41564.240	41674.354	41607.645	0.676	<0.001	<0.001
3C	-20614.118	41286.237	41438.299	41346.177	0.780	0.0031	<0.001
4C	-20494.130	41062.260	41256.270	41138.735	0.738	<0.001	<0.001
5C	-20415.567	40921.134	41157.092	41014.144	0.766	0.0025	<0.001
**6C**	**-20334.922**	**40775.844**	**41053.750**	**40885.389**	**0.812**	**0.0005**	**<0.001**
7C	-20253.471	40628.941	40948.796	40755.021	0.790	0.2268	<0.001

Note. LL, Log Likelihood; AIC, Akaike Information Criteria; BIC, Bayesian Information Criterion; aBIC, sample size-adjusted BIC; LMR(P), the p-value of the Lo-Mendell-Rubin test; BLRT(P), the p-value of the Bootstrapped likelihood ratio test; 1C, one-class; 2C, two-class; 3C, three-class; 4C, four-class; 5C, five-class; 6C, six-class; 7C, seven-class.

Table S6. Model selection criteria for the GMM analysis in the third imputation dataset.

Class	LL	AIC	BIC	aBIC	Entropy	LMR(P)	BLRT(P)
Free Estimation							
1C	-21204.881	42431.761	42489.440	42454.497	-	-	-
2C	-20686.132	41410.263	41509.890	41449.534	0.698	0.0005	<0.001
3C	-20501.645	41057.289	41198.864	41113.095	0.640	0.0060	<0.001
4C	-20350.289	40770.578	40954.101	40842.919	0.700	<0.001	<0.001
5C	-20253.671	40593.343	40818.814	40682.219	0.694	0.0002	<0.001
**6C**	**-20196.371**	**40494.742**	**40762.161**	**40600.153**	**0.721**	**<0.001**	**<0.001**
7C	-20135.278	40388.556	40697.923	40510.502	0.716	0.3634	<0.001
Linear Estimation							
1C	-21256.895	42531.789	42578.981	42550.391	-	-	-
2C	-20865.978	41761.956	41840.609	41792.959	0.617	<0.001	<0.001
3C	-20753.137	41548.274	41658.388	41591.679	0.649	<0.001	<0.001
4C	-20711.488	41476.976	41618.551	41532.782	0.706	0.0262	<0.001
5C	-20681.918	41429.836	41602.872	41498.044	0.715	0.0024	<0.001
6C	-20585.439	41249.098	41453.595	41329.707	0.776	0.0284	<0.001
**7C**	**-20566.430**	**41222.860**	**41458.818**	**41315.870**	**0.791**	**0.0270**	**0.0128**
8C	-20498.621	41099.242	41366.662	41204.654	0.834	0.3018	0.0128
Quadratic Estimation							
1C	-21193.421	42412.842	42481.007	42439.711	-	-	-
2C	-20765.682	41573.365	41683.479	41616.769	0.676	<0.001	<0.001
**3C**	**-20617.128**	**41292.256**	**41444.318**	**41352.195**	**0.781**	**0.0025**	**<0.001**
4C	-20496.464	41066.927	41260.937	41143.402	0.739	<0.001	<0.001
5C	-20418.410	40926.819	41162.777	41019.829	0.765	0.0025	<0.001
6C	-20342.936	40791.871	41069.777	40901.416	0.787	0.1319	<0.001

Note. LL, Log Likelihood; AIC, Akaike Information Criteria; BIC, Bayesian Information Criterion; aBIC, sample size-adjusted BIC; LMR(P), the p-value of the Lo-Mendell-Rubin test; BLRT(P), the p-value of the Bootstrapped likelihood ratio test; 1C, one-class; 2C, two-class; 3C, three-class; 4C, four-class; 5C, five-class; 6C, six-class; 7C, seven-class; 8C, eight-class.

Table S7. Model selection criteria for the GMM analysis in the fourth imputation dataset.

Class	LL	AIC	BIC	aBIC	Entropy	LMR(P)	BLRT(P)
Free Estimation							
1C	-21203.712	42429.424	42487.103	42452.160	-	-	-
2C	-20682.275	41402.550	41502.177	41441.821	0.696	<0.001	<0.001
3C	-20500.260	41954.520	41196.094	41110.326	0.679	0.0008	<0.001
**4C**	**-20361.823**	**40793.646**	**40977.169**	**40865.987**	**0.708**	**<0.001**	**<0.001**
5C	-20246.119	40578.239	40803.710	40667.115	0.717	0.0951	<0.001
Linear Estimation							
1C	-21256.067	42530.133	42577.325	42548.735	-	-	-
2C	-20886.403	41762.807	41841.460	41793.810	0.617	<0.001	<0.001
3C	-20753.311	41548.621	41658.735	41592.026	0.648	<0.001	<0.001
4C	-20710.601	41475.201	41616.776	41531.007	0.707	0.0462	<0.001
5C	-20681.539	41429.078	41602.114	41497.286	0.715	0.0004	<0.001
6C	-20644.672	41367.343	41571.840	41447.952	0.761	0.0223	<0.001
**7C**	**-20564.939**	**41219.878**	**41455.836**	**41312.888**	**0.794**	**0.0172**	**<0.001**
8C	-20491.927	41085.855	41353.274	41191.266	0.837	0.5722	0.2857
Quadratic Estimation							
1C	-21192.466	42410.933	42479.099	42437.802	-	-	-
2C	-20764.990	41571.979	41682.093	41615.384	0.676	<0.001	<0.001
**3C**	**-20618.136**	**41294.273**	**41446.335**	**41354.213**	**0.781**	**0.0063**	**<0.001**
4C	-20496.925	41067.849	41261.859	41144.324	0.739	<0.001	<0.001
5C	-20417.539	40925.078	41161.036	41018.088	0.766	0.0013	<0.001
6C	-20339.547	40785.094	41063.000	40894.639	0.785	0.0739	<0.001

Note. LL, Log Likelihood; AIC, Akaike Information Criteria; BIC, Bayesian Information Criterion; aBIC, sample size-adjusted BIC; LMR(P), the p-value of the Lo-Mendell-Rubin test; BLRT(P), the p-value of the Bootstrapped likelihood ratio test; 1C, one-class; 2C, two-class; 3C, three-class; 4C, four-class; 5C, five-class; 6C, six-class; 7C, seven-class; 8C, eight-class.

Table S8. Model selection criteria for the GMM analysis in the fifth imputation dataset.

Class	LL	AIC	BIC	aBIC	Entropy	LMR(P)	BLRT(P)
Free Estimation							
1C	-21203.078	42428.156	42485.835	42450.892	-	-	-
2C	-20684.035	41406.070	41505.697	41445.341	0.695	<0.001	<0.001
3C	-20501.090	41056.180	41197.755	41111.986	0.641	0.0101	<0.001
**4C**	**-20346.162**	**40762.324**	**40945.847**	**40834.665**	**0.703**	**0.0001**	**<0.001**
5C	-20250.671	40587.342	40812.813	40676.218	0.716	0.3240	<0.001
Linear Estimation							
1C	-21255.750	42529.500	42576.692	42548.102	-	-	-
2C	-20863.424	41756.848	41835.501	41787.851	0.619	<0.001	<0.001
3C	-20750.763	41543.525	41653.639	41586.930	0.650	<0.001	<0.001
4C	-20708.823	41471.646	41613.221	41527.452	0.708	0.0294	<0.001
5C	-20680.405	41426.810	41599.846	41495.017	0.713	0.0013	<0.001
**6C**	**-20661.218**	**41400.435**	**41604.932**	**41481.044**	**0.744**	**<0.001**	**0.0444**
7C	-20558.166	41206.333	41442.291	41299.343	0.786	0.0812	<0.001
Quadratic Estimation							
1C	-21191.993	42409.987	42478.153	42436.856	-	-	-
2C	-20763.223	41568.446	41678.560	41611.851	0.676	<0.001	<0.001
**3C**	**-20616.291**	**41290.581**	**41442.643**	**41350.521**	**0.781**	**0.0078**	**<0.001**
4C	-20495.022	41064.043	41258.053	41140.518	0.739	<0.001	<0.001
5C	-20417.304	40924.608	41160.566	41017.618	0.764	0.0029	<0.001
6C	-20341.238	40788.476	41066.382	40898.021	0.784	0.1805	<0.001

Note. LL, Log Likelihood; AIC, Akaike Information Criteria; BIC, Bayesian Information Criterion; aBIC, sample size-adjusted BIC; LMR(P), the p-value of the Lo-Mendell-Rubin test; BLRT(P), the p-value of the Bootstrapped likelihood ratio test; 1C, one-class; 2C, two-class; 3C, three-class; 4C, four-class; 5C, five-class; 6C, six-class; 7C, seven-class.

Table S9. Variables and coefficients selected by LASSO.

Variables	Class 1	Class 2	Class 3	Max abs coefficient
Age	-0.0491	0	0	0.0491
Education: other	0.2593	0	0	0.2593
Residence: rural	-0.5564	0	0	0.5564
BMI: 23 to 24.9	0.1469	0	0	0.1469
BMI: 25 to 100	-0.1944	0	0	0.1944
Grip strength	0.0316	0	-0.0003	0.0316
Waist	-0.0046	0	0	0.0046
HbA1c	-0.0511	0	0	0.0511
TC	-0.0009	0.0008	0	0.0009
Glucose	-0.0014	0.0006	0	0.0014
BUN	0	0	0.0093	0.0093
Creatinine	0.1066	0	0	0.1066
UA	0.0244	-0.0044	0	0.0244
Work status: workless	-0.2448	0	0.2148	0.2448
Smoking: yes	-0.1693	0	0	0.1693
Drinking: yes	-0.0567	0	0.0142	0.0567
Childhood health	-0.0648	0	0	0.0648
Death of children: yes	-0.4583	0.0063	0	0.4583
Life satisfaction	0.2701	0	-0.0900	0.2701

Note. BMI, body mass index; HbA1c, glycated hemoglobin; TC, total cholesterol; BUN, blood urea nitrogen; UA, uric acid; Class 1, Low-increasing; Class 2, High-fluctuating; Class 3, Elevated-stable.
